# A survey of medical students to assess their exposure to and knowledge of renal transplantation

**DOI:** 10.1186/1472-6920-4-32

**Published:** 2004-12-23

**Authors:** Anusha G Edwards, Andrew R Weale, Justin D Morgan

**Affiliations:** 1 Department of General Surgery, Southmead Hospital, Westbury on Trym, Bristol BS10 5NB, UK

## Abstract

**Background:**

Within the field of renal transplantation there is a lack of qualified and trainee surgeons and a shortage of donated organs. Any steps to tackle these issues should, in part, be aimed at future doctors.

**Methods:**

A questionnaire was distributed to final year students at a single medical school in the UK to assess their exposure to and knowledge of renal transplantation.

**Results:**

Although 46% of responding students had examined a transplant recipient, only 14% had ever witnessed the surgery. Worryingly, 9% of students believed that xenotransplantation commonly occurs in the UK and 35% were unable to name a single drug that a recipient may need to take.

**Conclusions:**

This survey demonstrates a lack of exposure to, and knowledge of, the field of renal transplantation. Recommendations to address the problems with the recruitment of surgeons and donation of organs, by targeting medical students are made.

## Background

With the potential for improved quality of life and increased life expectancy, renal transplantation is the first choice treatment for most patients with end-stage renal failure [1,2]. However, in the UK there is an ever-increasing disparity between the number of patients on the waiting list and those being transplanted [3]. This is predominantly due to a rise in the incidence of renal failure amongst an aging, racially diverse society in conjunction with a shortage of donated organs [4].

The field of renal transplantation also suffers from a lack of surgeons. Indeed, it is predicted that by the year 2005 there will a shortage of over twenty consultant renal transplant surgeons [5].

Any measures to deal with these problems must include educating and attracting the doctors of tomorrow; medical students [6,7]. However, the General Medical Council's core curriculum model for undergraduate teaching has lead to significant changes in the way that specialist subjects are taught [8]. This survey was conducted to assess the exposure to, and knowledge of, renal transplantation amongst medical students at a single medical school in the United Kingdom.

## Methods

**Figure 1:**
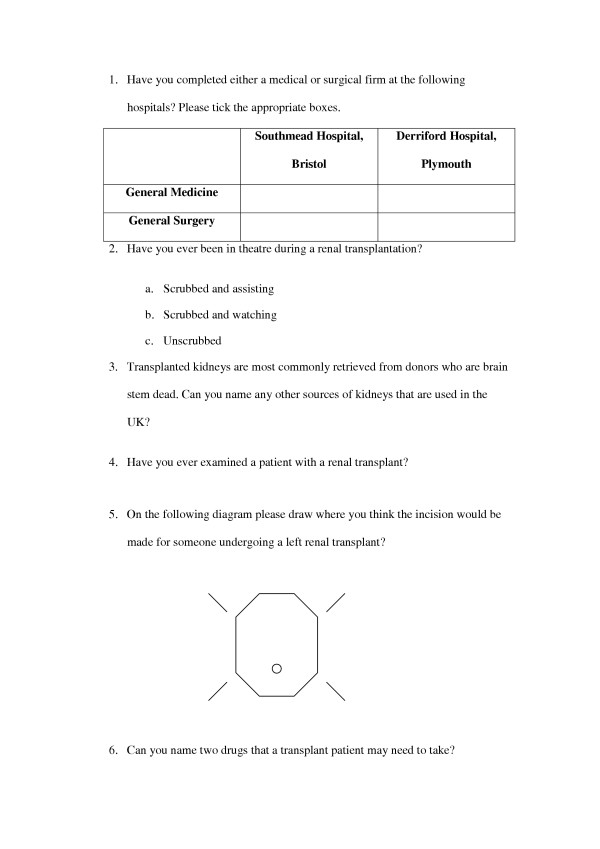
Questionnaire distributed to the students.

In July 2003 a PRHO job fair was held for final year medical students of Bristol University, which all 140 students within the year attended. An anonymous questionnaire was distributed to every student, after the aims of the study had been explained, at the end of a series of short lectures. (Figure 1). The questionnaire consisted of six questions, three to assess their exposure to the field and three to assess their knowledge. The form was collected immediately upon completion, with no opportunity to confer.

The questionnaire had been piloted on a random sample of thirty final year medical students from the year before, whereby the form was distributed electronically to their University email accounts. This confirmed that the length and wording of the questionnaire, and the level of knowledge required to complete it were appropriate for the target population.

All statistical analysis was performed using the chi square test, with a p value of 0.05 or less taken to demonstrate statistical significance.

## Results

Questionnaires were completed by seventy-six of the 140 medical students in the year (54%). Of these responding students, thirty-two (42%) had never completed either a general medical or general surgical placement at one of the two centres for renal transplantation within the region, both of which are affiliated to the University of Bristol. National data of renal transplantation activity shows that these two centres differ in the number of transplants performed each year [3]. Over the 2002/3 and 2003/4 period Southmead Hospital, Bristol performed approximately three times as many transplants per year than Derriford Hospital, Plymouth (2002/3: 101 in Bristol, 36 in Plymouth, 2003/4: 132 in Bristol, 42 in Plymouth).

Sixty-five students (86%) had never been in the operating theatre during a renal transplant. Of the eleven students that had witnessed a transplant the majority, seven, had been unscrubbed (9%). One student had been scrubbed and observing and a further three students (4%) had actually assisted with the procedure. Closer analysis of these eleven students demonstrates that only one of the eleven had been on placement at Derriford Hospital (χ^2^, p = 0.006).

Thirty-five (46%) of the students had examined a patient with a transplanted kidney but only thirty-three (43%) could accurately draw on a diagram the usual site of surgical incision that would be made on someone undergoing a left sided renal transplant. Interestingly, fourteen of the students that claimed to have examined a transplant recipient were unable to accurately draw the site of incision. Of the students that had examined a transplant recipient twenty-two had been on placement at one of the hospitals with transplant centres and thirteen had not (χ^2^, p = 0.128).

Fifty students (66%) were aware that in addition to the use of organs from brain stem dead donors, kidneys could also be transplanted from living donors. Eighteen students (24%) could not name any additional sources of organs and seven (9%) thought that xenotransplantation, using porcine kidneys, is carried out in the UK. None of the students suggested non-heart beating donation.

Nineteen students (25%) were unable to name a drug that might be taken by a patient with a renal transplant. Twenty-one students (28%) were able to suggest just one.

## Discussion

This survey highlights both a low exposure to, and a lack of knowledge about, the field of renal transplantation amongst medical students. This is cause for concern as it has implications for the future recruitment of trainees to the speciality and, potentially, to the procurement of organs.

Previous work has highlighted the multiple factors that deter surgical trainees from this speciality [5]. These include the on-call commitment, unpredictable workload and a lack of exposure to the speciality, at an early stage in training. Based on this information, calls have been made to increase the exposure of surgical trainees by the inclusion of transplantation within basic surgical training (BST) rotation programmes [5]. However, as there are only 23 surgical centres performing renal transplantation within the UK it is unlikely that all trainees would be exposed to the field. In order to gain exposure to the maximum number of doctors, early in their careers, targeting medical students may yield the best results. Indeed, a recent crisis meeting regarding recruitment to renal transplant surgery identified that early positive exposure to the field is vital, and should begin at the undergraduate level [6]. This survey highlights that the potential for the promotion of renal transplantation within the undergraduate course is currently relatively unexplored.

The lack of knowledge regarding sources of organs commonly used within the UK is also of concern. In order to increase the number of kidneys available UK Transplant funds a number of non-heart beating programmes. Such initiatives can potentially increase the transplant rate by 20–40% [9]. Identifying all potential heart beating and non-heart beating donors is fundamental to providing a successful service, and reducing the gap between donors and patients on the waiting list. However, if future doctors are unaware of the existence of these programmes then such schemes are unlikely to reach their full potential.

One of the limitations of this study is the selection bias from a 54% response rate. From talking to some of the students who did not complete the questionnaire it became apparent that those who had no experience or knowledge of the speciality were less likely to participate. This means that the results are probably over reporting the exposure to and knowledge of renal transplantation. A further limitation is that this work only represents the situation at one medical school. We believe that this situation is not unique to Bristol University and recommend that a national study be performed to assess the true extent of the situation.

If transplantation rates are to be maximised and recruitment into the speciality improved, then ideally, all doctors should have some exposure to renal transplantation during the early stages of their career. Indeed a recent study has demonstrated that increased knowledge about organ donation is associated with higher levels of medical education, an increased likelihood of holding an organ donor card and feeling more comfortable in approaching relatives of potential organ donors [7]. Whilst it is realised that teaching time is of a premium at medical school this would be the ideal opportunity to promote transplantation. We believe that conventional methods such as ward based teaching, lectures and tutorials could be supplemented with a more multidisciplinary exposure. For example, involving students in patient education open days or the production of information leaflets/web pages could allow students to see from themselves the improvement in quality of life brought about by transplantation; Such learning experiences may prove more memorable for some students than those of the operating theatre.

## Conclusions

This survey, carried out at a single UK medical school, has highlighted a low level of exposure to, and a lack of knowledge about, renal transplantation amongst medical students. These worrying results may influence the outcomes of any measures put in place to improve the recruitment of surgeons and the procurement of organs. If the trends within these areas are to be reversed then greater emphasis should be placed upon the promotion of renal transplantation within the undergraduate curriculum.

## Abbreviations

**UK **United Kingdom

**PRHO **Pre-registration house officer

## Competing interests

The author(s) declare that they have no competing interests.

## Authors' contributions

AGE designed the questionnaire, performed the study and was involved in the preparation of the manuscript. ARW and JDM were also involved in the design of the questionnaire and manuscript preparation.
